# Capitalizing on Implementation Science to Advance Antimicrobial Stewardship and Health Equity in Treating Pediatric UTI’s

**DOI:** 10.1017/ash.2024.107

**Published:** 2024-09-16

**Authors:** Holly Maples, Jessica Snowden, Valerie Harfield, Geoff Curran

**Affiliations:** Uams Cop; University of Arkansas for Medical Sciences; Arkansas Childrens

## Abstract

**Background:** Pediatric urinary tract infections represent the most common pediatric infection with increasing gram-negative antibiotic resistance. Overutilization of antimicrobials including third generation cephalosporins are known drivers of this resistance. Antimicrobial stewardship (AS) efforts have recently shown that antibiotic selection may be influenced by patient race. Implementation science (IS) can provide frameworks and strategies to improve antimicrobial utilization and equity. **Methods:** This was a pre/post study of 2 geographically different children’s hospitals general pediatric floors assessing the impact of a set of implementation strategies developed to improve provider knowledge of best practice antimicrobials (based on local susceptibilities for treatment of UTI’s) and influence uptake of best practice prescribing. IS strategies included provider education, local clinical champion and opinion leader involvement, leadership involvement, local policy changes, and stakeholder co-design of decision support tools (a clinical pathway, a specific ITI antibiogram, and a dynamic order set). No education was provided regarding racial differences in prescribing habits. Outcomes were measured utilizing a portion of the RE-AIM Framework of assessing adoption of “Right” antibiotic, order set adoption, and equitable reach (racial differences in prescribing). **Results:** Hospital A and B had a first-generation cephalosporin prescription rate of 29.7% (n=441) and 20.6% (n-557) pre implementation and 44.6% (n=84) and 47.5% (n=118) post (p < 0 .001). Both hospitals also saw a significant reduction in third-generation cephalosporins. In Hospital A, APRN’s were more likely to prescribe a first generation cephalosporin (52.4%) than a DO (42.1%) or MD (26.4%) pre-implementation (p=0.004). In Hospital B, APRN’s were less likely to prescribe first generation cephalosporins (5.4%) than a DO (28.9%) or MD (19.4%) pre-implementation (p=0.004). No statistical significance was seen post implementation for antimicrobial selection by provider type for either hospital. Based on race, both hospitals had Black and Other patients receiving more first-generation cephalosporins while white patients were more likely to receive third-generation cephalosporins (p=0.033) pre implementation. No statistical significance was seen post implementation for antimicrobial selection based on race. No improvement was seen in order set utilization. **Conclusion:** With order set utilization not improving with implementation of new dynamic order set, other strategies such as education, clinical champion and opinion leader involvement, and provision of a local UTI antibiogram were likely contributors to the improvement in best antimicrobial for treatment of UTI’s. Further mixed method research is warranted to improve understanding of the relative performance of our strategies, especially the lack of provider adoption of the novel dynamic order set.

**Disclosure:** Jessica Snowden: Advisory board - Pfizer

